# The music of morality and logic

**DOI:** 10.3389/fpsyg.2015.00908

**Published:** 2015-07-01

**Authors:** Bruno Mesz, Pablo H. Rodriguez Zivic, Guillermo A. Cecchi, Mariano Sigman, Marcos A. Trevisan

**Affiliations:** ^1^Department of Science and Technology, National University of QuilmesBuenos Aires, Argentina; ^2^Computation Department, University of Buenos AiresBuenos Aires, Argentina; ^3^Biometaphorical Computing, Thomas J. Watson Research Center, IBMYorktown Heights, NY, USA; ^4^Integrative Neuroscience Lab, Physics Department, University of Buenos Aires-IFIBA, Consejo Nacional de Investigaciones Científicas y TécnicasBuenos Aires, Argentina; ^5^Business School, Universidad Torcuato Di TellaBuenos Aires, Argentina; ^6^Dynamical Systems Lab, Physics Department, University of Buenos Aires-IFIBA, Consejo Nacional de Investigaciones Científicas y TécnicasBuenos Aires, Argentina

**Keywords:** morality, logic, semantic content, musical structure, music psychology

## Abstract

Musical theory has built on the premise that musical structures can refer to something different from themselves (Nattiez and Abbate, 1990). The aim of this work is to statistically corroborate the intuitions of musical thinkers and practitioners starting at least with Plato, that music can express complex human concepts beyond merely “happy” and “sad” (Mattheson and Lenneberg, 1958). To do so, we ask whether musical improvisations can be used to classify the semantic category of the word that triggers them. We investigated two specific domains of semantics: morality and logic. While morality has been historically associated with music, logic concepts, which involve more abstract forms of thought, are more rarely associated with music. We examined musical improvisations inspired by positive and negative morality (e.g., good and evil) and logic concepts (true and false), analyzing the associations between these words and their musical representations in terms of acoustic and perceptual features. We found that music conveys information about valence (good and true vs. evil and false) with remarkable consistency across individuals. This information is carried by several musical dimensions which act in synergy to achieve very high classification accuracy. Positive concepts are represented by music with more ordered pitch structure and lower harmonic and sensorial dissonance than negative concepts. Music also conveys information indicating whether the word which triggered it belongs to the domains of logic or morality (true vs. good), principally through musical articulation. In summary, improvisations consistently map logic and morality information to specific musical dimensions, testifying the capacity of music to accurately convey semantic information in domains related to abstract forms of thought.

## Introduction

Reason and emotion span a large portion of culture which of course includes music perception (Krumhansl, 2002). Modern neuroscience has re-evaluated the interaction between these two aspects of mental activity (Damasio, 1995), particularly the extent of their overlap in moral judgment (Haidt, 2001). Increasing evidence indicates that cognitive and emotional brain structures are co-activated during the evaluation of morally laden situations (Greene et al., 2001; Decety et al., 2012; Koster-Hale et al., 2013).

The domain of cultural concepts related to virtues and morality has been associated with music since Antiquity. Ever since, music theorists have proposed correspondences between specific musical structures and moral features (Friedlein, 1867; Plato, 2002), and have sought explanations for the effect of music in moral emotions and behavior (Kivy, 2009). Recent experimental evidence shows that disgusting and irritating sounds, and instrumental music expressing anger or happiness, can have significant impact in moral judgments (Seidel and Prinz, 2013a,b). Morality therefore provides a unique vantage point from which to appraise the communication capacity of music: can musical structures consistently convey moral concepts?

An even more intriguing question is whether music can convey logical concepts, which are associated with abstract reasoning. Based on the results mentioned above, which demonstrate an interaction between logic and moral representations in human judgments (beyond the domain of music), one may hypothesize that music has the ability to refer semantically to the category of logic.

Here we sought to statistically corroborate the intuitions of musical thinkers that music can express complex human concepts beyond merely “happy” and “sad.” To this aim, we study free music improvisations inspired by positive and negative moral concepts, e.g., good and evil, and positive and negative logic concepts, e.g., true and false. There is of course some degree of transfer between the semantic domains of logic and morality, which reflects a classic philosophical problem about the relationship between beauty, goodness, and truth.

Our aim is to investigate the capacity of music to convey this fine semantic distinctions by analyzing the musical improvisations to words pertaining to these categories. We show that music represents reliably semantic information, coding through different acoustic and perceptual features the category and the valence of a given word.

## Results

We selected four groups of eight words related to positive logic (e.g., exactness), negative logic (e.g., inexactitude), positive morality (e.g., charity) and negative morality (e.g., avarice). Negative moral concepts were obtained from the capital sins (except “evil” which we included to form a set of eight words). The representatives of the other categories were selected in the following way. Operationally, we choose the three basic concepts “truth,” “falsehood,” and “goodness.” The corresponding Wikipedia entry for each word is then considered as a bag of words, and ranked by frequency of occurrence. From this list, we finally choose the top eight abstract nouns (i.e., discarding concrete nouns, adjectives, verbs, etc.), resulting in the following list:

- *Positive logic*: truth, certainty, consistency, exactness, adequacy, authenticity, conformity, and reality.
- *Negative logic*: falsehood, inexactitude, incorrectness, inadequacy, error, doubt, deception, and misrepresentation.
- *Positive morality*: loyalty, goodness, charity, solidarity, humility, generosity, prudence, and patience.
- *Negative morality*: lust, gluttony, laziness, evil, avarice, envy, pride, and anger.

These concepts may have an overlapping mental representation, such as may be the case, for instance, with “good” and “truth.” A significant overlap may be a confounding factor in our analysis, as the ability of music to convey concepts associated with “good” could explain why it can also convey information related to “truth.” To verify the correspondence of the words with their categories, we ran a control experiment (12 subjects) where participants classified the 32 words to each category. Results showed a consistent association of exemplars with their corresponding categories: error rates were below 4% for all categories. Even for the word showing the lowest score of assignment agreement (deception), the correspondence was quite accurate (66%) with 4 out of 12 subjects assigning these words to negative morality instead of to negative logic. We did not observe any mismatch between categories of different valences. We further submitted our list of words to an LSA analysis, which creates a vectorial representation of concepts based on their frequency of co-occurrence in large text corpora, in such a way that the semantic relatedness of concepts can be estimated by the proximity of their respective vectors. Following the analysis detailed in the Supplementary Materials, our results indicate that, to the extent that semantic relatedness is captured by LSA, the overlap across concept classes is minimal.

We then presented visually each of the 32 words in randomized order to 19 professional pianists. The musicians were asked to freely improvise in a MIDI keyboard a piece evoked by the presented word. This lead to a set of 608 MIDI files (32 words × 19 musicians) and corresponding sound files obtained from them using the software Timidity (see Methods).

**Figure 1:**
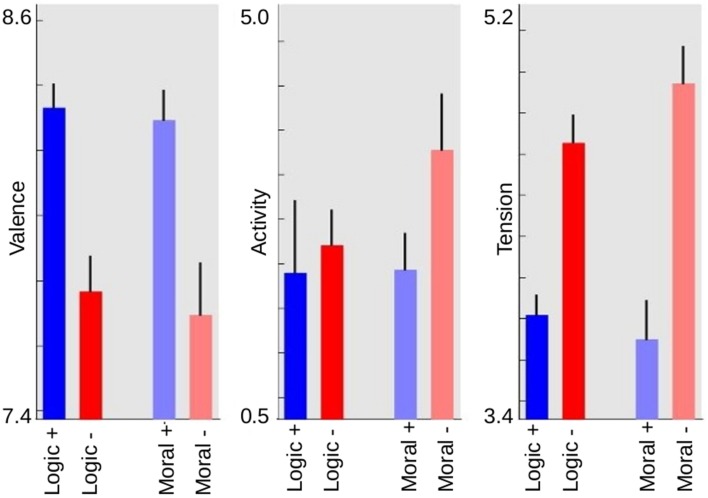
**Musical emotion space**. We used the MIRToolbox model of emotion rating of music audio files in the three-dimensional space of valence, activity, and tension (Eerola et al., 2009). The model uses multiple linear regression with five acoustic predictors for each emotional dimension. MIDI files were converted to audio files using Timidity. As expected, semantic valence reliably maps to valence in the musical space. Activity was affected by moral valence (good vs. evil) but not by logic valence (true vs. false), and tension was affected by semantic valence (true and good vs. false and evil) but not by semantic category (good and evil vs. true and false).

To validate the quality of the improvisations produced by the 19 professional pianists (and to confirm the representativeness of the words chosen for each semantic category) we used standard algorithms to rank the emotional content of each music file. Specifically, we used a model to rate three emotional dimensions (valence, activity, and tension) based on a large sample of empirical data of affective labeling by listeners. Full details of this algorithm are provided in the Methods Section and in (Eerola et al., 2009), but in essence it is based on subjective ratings of a collection of soundtracks, which were then fitted using Multiple Linear Regression on a set of acoustic and psychoacoustic features (such as energy of the signal and spectrum center). As a first step to test the reliability of our data, we verified that improvisations based on words associated with positive logic and morality had greater musical valence than words associated with negative logic and morality. This highly expected result merely says that valence in the domain of semantics maps reliably to valence in the relevant musical dimensions of the improvisations. We measured the valence of each music file and then submitted the data to an ANOVA analysis with subjects as a random factor and semantic valence (positive or negative) and semantic category (morality or logic) as within-subject factors. Results (Figure 1, left panel) showed a clear effect of semantic valence (*df* = 1, *F* = 33.8, *p* < 0.001) and no effect of semantic category (*df* = 1, *F* = 0.45, *p* > 0.5) nor an interaction (*df* = 1, *F* = 0.14, *p* > 0.7). Similarly, an ANOVA analysis of musical tension (Figure 1, right panel) showed an effect of semantic valence (*df* = 1, *F* = 31.73, *p* < 0.001) and no effect of semantic category (*df* = 1, *F* = 0.39, *p* > 0.5) nor an interaction (*df* = 1, *F* = 0.35, *p* > 0.5). The effects were large; words associated with negative logic or negative morality elicited improvisations with almost a full point increase in their perceived tension (Figure 1, right panel). An analysis of activity showed instead that it was affected by moral valence, but not by logic valence; words with positive or negative logic yielded improvisations with comparable perceived emotional activity (Figure 1, central panel). This was confirmed by an ANOVA analysis which showed a main effect of valence (*df* = 1, *F* = 18.05, *p* < 0.001), no effect of semantic category (*df* = 1, *F* = 2.94, *p* > 0.1), and contrary to the other emotional parameters, a highly significant interaction between semantic valence and semantic category (*df* = 1, *F* = 35.6, *p* < 0.001).

Our main aim was to rank different dimensions of music in their ability to communicate semantic valence (positive or negative) and semantic category (morality and logic). To this aim we computed, for each music file, a set of 12 parameters that represent musical dimensions. We then encode each music file as a 12 dimensional vector. The 12 musical dimensions can be grouped in three main families:

(i) **Roughness** (or sensorial dissonance) which quantifies the beating frequencies, (ii) **brightness**, the proportion of high-frequency spectral energy of the sound, and (iii) **attack time**, the time to reach maximum sound intensity of the note. These dimensions correspond to the family of *acoustical parameters* which characterize the timbre of complex sounds (Caclin et al., 2005).
(iv) Pitch **entropy**, that measures the existence of locally dominant pitches and correlates with the predictability of a musical composition at different temporal scales, considering its repetitions and internal symmetries (Eerola et al., 2002) and (v) **melodic entropy**, which considers only the pitches of the highest voice (that usually contains the melody); these measures belong to the family of *information complexity parameters*.
(vi) **Ambitus**, the difference between the highest and the (vii) **lowest note** on a composition; (viii) average note **duration**; (ix) **articulation**, which measures the degree of continuity between successive notes, from almost no pause between notes (*legato*) to detached notes (*staccato*); (x) **velocity**, related to the loudness and sound volume of the improvisation; (xi) **dissonance** and (xii) **gradus**, used to determine the degree of harmonic and melodic dissonance respectively. Dissonant music is judged as unpleasant or unstable while consonant music is typically associated with pleasantness. These form the set of *compositional parameters* (Mesz et al., 2011).

These 12 parameters span the constitutive elements of music, from its sound substrate (1.) to its more abstract temporal pitch organization and compositional structure (2. and 3.).

Once the musical pieces were projected to this parameter space, we analyzed which dimensions are more effective to discriminate between valences (positive and negative) and between semantic categories (moral and logic).

**Figure 2:**
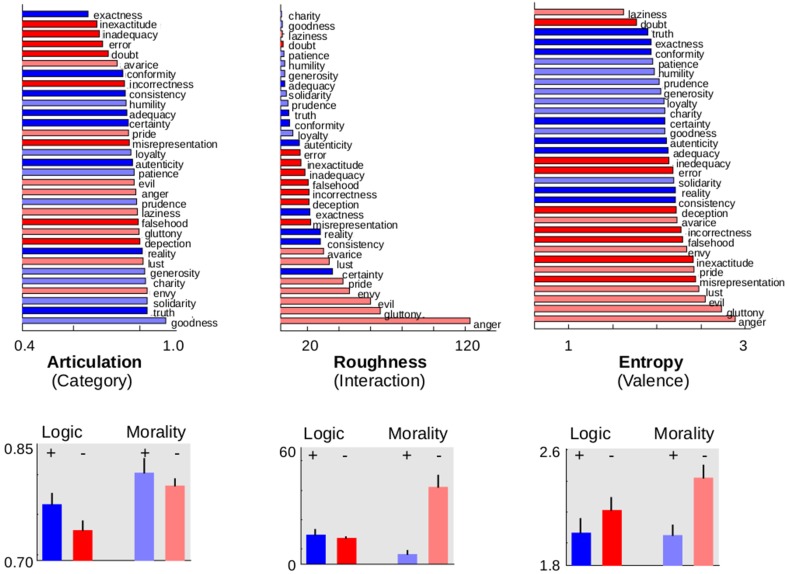
**Musical, structural, and acoustical parameters for morality/logic concepts**. Organization of musical improvisations for three parameters, where blue codes improvisations elicited by positive logic words, light blue for positive morality, red for negative logic, and light red for negative morality. The upper panels show the relative ordering of the presented words, averaged across pianists. The lower panels show mean values and standard deviations averaged across words and pianists. *Articulation* (left panels) is the only dimension to significantly discriminate the morality and logic categories (light from dark tones). This parameter varies between 0 (for *staccato*, notes separated by silences) and 1 (for *legato*, no breaks between notes). *Roughness* (middle panels) is the only dimension showing a significant interaction, discriminating valence for morality and not for logic words. Its range goes from 0 for a pure sine wave (no partial beats) to over 400 for white noise. On the other hand, *entropy* (right panels) is one of the parameters that significantly discriminate valence (blues from reds). Entropy of a single repeated note is 0 and its value increases for uniformly distributed notes. The mean values of the rest of the parameters can be found at Table 1 and Figure S2.

To this aim, we first submitted the values of each parameter to an independent ANOVA analysis with subjects as a random factor and valence (positive or negative) and semantic category (morality or logic) as within-subject factors. The resulting *p*-values of each ANOVA were corrected with a strict Bonferroni criterion to account for multiple comparisons and hence only *p*-values lower than 0.05/12 = 0.0041 were considered as significant. Analyses revealed that 7 out of the 12 dimensions corresponding to the three families distinguished positive from negative valence (see Table 1), indicating that valence information is widely distributed along musical properties. Positive valence was associated with higher scores of **attack time** and **lower note** and with lower scores of **roughness, ambitus**, **dissonance**, **entropy**, and **melodic entropy** compared to negative valence (see right panels of Figure 2 and Figure S1).

**Table 1:** **ANOVA analysis with valence (positive and negative) and domain (morality and logic) as independent factors**.

**Parameter**	**Word valence**		**Word domain**		**Interaction**	
	***F***	***p***	***F***	***p***	***F***	***p***
Ambitus	12.39	**0.0024**	0.05	0.82	6.27	0.012
Lowest note	18.34	**0.0004**	0.07	0.79	6.93	0.008
Duration	5.05	0.037	2	0.17	0.12	0.73
Velocity	6.33	0.021	0.57	0.46	1.53	0.21
Articulation	2.85	0.1086	16.73	**0.0007**	0.24	0.621
Gradus	4.61	0.0455	0.53	0.47	2.6	0.1076
Dissonance	11.78	**0.0029**	1.47	0.24	2.6	0.1076
Entropy	14.98	**0.0011**	4.8	0.0416	6.45	0.0114
Mel. entropy	18.39	**0.0004**	0.81	0.38	0.44	0.5
Roughness	27.54	**0.0001**	8.31	0.0095	54.02	0
Brightness	2.44	0.13	3.55	0.07	0.06	0.8
Attack time	11.52	**0.0031**	0.57	0.45	8.09	0.0046

*The analysis revealed that word valence has a significant effect in seven parameters, while word domain presents a significant effect on articulation. An interaction effect was present on roughness. A cutoff point of p < 0.004 is noted in bold type*.

The ANOVAS also revealed that musical dimensions were overall considerably less efficient to distinguish between categories (morality or logic). In effect, **articulation** was the only dimension to significantly discriminate morality from logic after multiple comparisons correction (Figure 2, left panels). A *post-hoc t*-test showed that this was accounted both by an effect of category for positive words (morality = 0.961 ± 0.004, logic = 0.903 ± 0.008, *t* = 4.28, *p* = 0.036, *df* = 7) and effect of category for negative words (morality = 0.902 ± 0.008, logic = 0.868 ± 0.006, *t* = 2.76, *p* = 0.028, *df* = 7), revealing that moral words produce more legato and logic words more staccato improvisations.

**Roughness** was the only dimension showing a significant interaction (Figure 2, central panels). *Post-hoc t*-test showed that this effect was accounted by a highly significant effect of valence for moral words (positive = 6.39 ± 1.3, negative = 51.11 ± 3.9, *t*-test = 3.74, *p* = 0.072, *df* = 7) while there was no effect of valence in the logic category (positive = 19.17 ± 2.45, negative = 17.65 ± 1.62, *t* = 0.27, *p* > 0.7, *df* = 7). Entropy showed a marginally significant interaction revealing the same trend as roughness which did not survive multiple comparisons (Figure 2, central panels).

The ANOVA analyses can identify which components discriminate between categories but it cannot quantify the classification precision nor inform on whether several dimensions can be combined to achieve a better classification. To answer these questions, we performed a classification-based analysis using a standard Machine Learning (ML) algorithm. ML classifiers train a model with a subset of the data and then compute the degree of predictability on the rest of the data.

The algorithm learns a model to relate concept classes to regions of the 12 dimensional musical space. This association is done in a group of subjects referred as *training set*. Then, the model takes an improvisation belonging to the complementary set of subjects, referred as *test group*. The model makes a prediction about the class to which the improvisation belonged from its musical parameters. This process is repeated changing the test and trained groups of subjects. From this analysis one can calculate the precision of the classification process compared to chance level which is 50% in all binary classifications. A substantial advantage of this algorithmic approach is that it is independent of the cultural and personal biases that would appear, for instance, in a listening and labeling task with human subjects trying to identify the domain and valence of the stimulus word just from the music.

**Figure 3:**
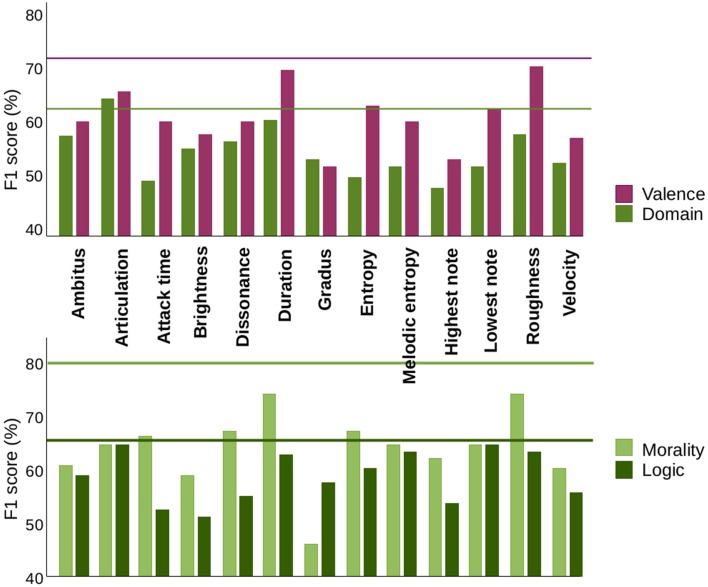
**Machine learning classifier**. A Machine Learning model was trained using the MIDI dataset of 15 pianists (480 improvisations) to predict the results of the other 4 pianists (for a total of 128 improvisations). The results shown are the average over all the combinations of training sets of 15 pianists and test sets of 4 pianists. Upper panel: *F*_1_score measuring predictability for discrimination of valences across domains (purple) and domains across valences (green). Lines indicate the joint performance of all parameters (fine-dashed for valence and dashed for category). Lower panel: *F*_1_ score measuring predictability for discrimination of positive from negative morality (light green) and positive from negative logic (dark green). Lines indicate the joint performance of all parameters.

We analyzed the predictive power of each individual parameter and of the complete set of 12 parameters. A standard way to measure the performance of a classifier is the *F*_1_ score (Alpaydin, 2009). Basically, it measures the percentage of correctly predicted results (see Methods). The pattern of classifying performance (Figure 3) is overall consistent with the ANOVA analyses. First, it shows consistently higher classification scores for valence than for category (Table 1 and Figure 3, upper panel) and also higher scores for valence restricted to moral words than to logic words (Table 2 and Figure 3, lower panel). Second, overall, the dimensions which showed discrimination in the ANOVA analyses showed high classification scores in the ML analysis. Roughness, which showed the highest significance values in the ANOVA analyses, also showed the greatest classification power (70.8 ± 0.4). One difference is duration, a strong carrier of valence in the classification analysis (68.9 ± 0.3) that did not reach significance in the ANOVA analysis after correction for multiple comparisons. These discrepancies are typical when the distribution has outliers: this severely affects the standard deviation (making ANOVA discrimination less significant) but does not have a strong impact in the classifiers.

**Table 2:** **ANOVA analysis of valence restricted to morality and logic**.

**Parameter**	**Valence on morality**		**Valence on logic**	
	***F***	***p***	***F***	***p***
Ambitus	28.45	**<10^−4^**	1.99	0.15
Lowest note	34.85	**<10^−4^**	5.44	0.0204
Duration	1.57	0.21	2.79	0.09
Velocity	4.21	0.041	0.14	0.7
Articulation	1.55	0.21	2.18	0.14
Gradus	0.92	0.33	7.63	0.0061
Dissonance	11.83	**0.0007**	3.04	0.08
Entropy	26.06	**<10^−4^**	4.31	0.0388
Mel. entropy	16.9	**<10^−4^**	10.82	**0.0011**
Roughness	54.47	**<10^−4^**	0.41	0.52
Brightness	0.3	0.58	1.36	0.24
Attack time	26.37	**<10^−4^**	1.92	0.16

*The analysis revealed that the valence restricted to morailty has a significant effect in the same parameters found in Table 1, while valence restricted to logic presents a significant effect on melodic entropy and a marginally significant effect on gradus. A cutoff point of p < 0.004 is noted in bold type*.

The main advantage of the ML analyses is that one can inquire whether different dimensions contribute synergistically to categorical classification or if, alternatively, the different dimensions provide redundant information. To test for synergy, we implemented a predictive model using as input the entire 12-dimensional vector representation of the parameters. Results showed that only the discrimination of morality concepts was synergetic, reaching an *F*_1_ score of 0.82 when all features were combined which is a significant (*p* < 0.001) increase over the best individual features, which reach ~0.75 as shown in Figure 3 (Duration and Roughness). In contrast, the discrimination of logic concepts does not display feature synergy (*p* > 0.1), as the best classifier reaches 0.65, comparable with the best individual feature (Articulation and Lowest Note, see Figure 3).

## Discussion

Here, we showed that despite individual variability in musical composition, musical improvisations can be used to accurately classify the valence of the word which triggers them and, to a lesser degree, also its semantic category.

Positive concepts are represented by music with regular and predictable structure, with low harmonic dissonance, low roughness and contained within a higher and comparatively narrower pitch range. Instead, articulation is the dimension with highest capacity to discriminate between logic and morality words. Logic words (regardless of valence) produce more staccato improvisations. Instead moral words, produce more legato improvisations, corresponding to more continuous music.

Our broad finding of a spontaneous associations of musical parameters to meaning fits well both with previous theoretical thinking of music and cognitive neuroscience studies in other domains of association.

### Musicology

From a theoretical perspective, the observed correspondences between musical features and the morality and logic domains can be interpreted from the notion of “meaning” in a broad sense, as the reference of musical structures to something different from themselves (Nattiez and Abbate, 1990). More generally, musical information has been interpreted with reference to extra-musical concepts throughout history (Plato, 2002; Zbikowski, 2005). This view, for which we provide evidence in this manuscript, is in tension with a prominent belief in linguistics that rejects the notion that music can transfer specific semantic concepts (Mattheson and Lenneberg, 1958).

More specific to the semantic categories investigated here, concepts related to virtues and morality have been associated with music since Antiquity. From this historical and theoretical perspective it is more surprising that logic concepts, usually associated with abstract logical reasoning, are musically represented, even if this representation achieves less classification power.

Our strategy in this study was to identify the dimensions conveying meaning using an automated analysis of musical parameters. This has the advantage of an objective evaluation of musical properties but it also has inconvenients which should be addressed in future studies. First, while some parameters lend themselves easily to machine analysis (e.g., ambitus, attack time, velocity), others may be less ideally suited for this type of inquiry—including dissonance, entropy, gradus, etc., all parameters which are frequently context-oriented, and convey tremendous semantic information. A second limitation which should be addressed in future studies is possible cultural variations of our results. All our pianists were from Argentina and with long trajectories of formal musical instruction. It would be of interest to understand which of our observations may vary in non-Western improvisational contexts. Would there be commonality in “roughness” or “brightness” between Chinese pianists (or erhu players) and the musicians used in this experiment?

### Cognitive neuroscience

The spontaneous production of reliable musical features from semantic categories fits well with previous experiments showing that cross-modal associations -often mediated by semantic representations- are ubiquitously present in normal cognitive function (Hubbard and Ramachandran, 2005; Cytowic and Eagleman, 2009; North, 2011). Moreover, music can be reliably related with meaning in other domains of semantics than logic and morality which we studied here. For instance, Koelsch and collaborators (Koelsch et al., 2004) showed that music can prime the meaning of a word and determine physiological indices of semantic processing (showing for instance that certain passages of Beethoven symphonies can prime the word “*hero*” rather than the word “*flea*”). Baraldi et al. (2006) performed an experiment in which musicians and non-musicians had to produce piano improvisations according to different semantic intentions. Listeners were able to recognize the majority of these intentions with very brief musical fragments.

To our knowledge, there are no previous investigations relating music and words belonging to morality and logic. However, more generally, brain imaging studies have shown that sensory and motor systems are similarly activated during conceptual and moral processing (Kiefer et al., 2008; Mahon and Caramazza, 2008; Lakens et al., 2011) constituting a precedent for the ubiquitous association between moral and logic words and musical forms. Similarly, there are spontaneous associations between moral words and other sensory representations: moral and immoral words are associated with up and down directions and white and black visual cues (Meier et al., 2007; Sherman and Clore, 2009).

### Musical dimensions conveying information about logic, morality and valence

Beyond the main result showing that music can convey information about valence and -with lower precision- about semantic category, our investigations sheds light on which specific dimensions of music are informative to convey these different aspects of semantics and how they synergize.

#### Emotion expression

It is important to note that in the following we focus on the recognition of emotion-related features in the music, which does not in itself entail the emotional response. Current evidence suggests that perceptions are typically stronger than feelings (Hunter and Schellenberg, 2010). In our study, improvisations elicited by words belonging to different groups contain significantly different acoustic cues for emotion: music inspired by positive moral words is more consonant than the one inspired by negative moral words. **Roughness** is eight times greater for negative moral than for positive moral words. Moreover, this single parameter classifies positive concepts with a correct rate of almost 0.75 in the machine learning model. These results are consistent with previous findings relating consonance and roughness to emotions. Dissonance correlates with activity in brain regions involved in processing negative stimuli (Blood et al., 1999) and more generally harmonic and sensory consonance versus dissonance and roughness have been shown to be consistent predictors of positive vs. negative emotional valence. High/low activity is associated to fast/slow tempos, and loud/soft, dissonant/consonant, and staccato/legato music. Tension is a more specifically musical emotional dimension; it is related to fast tempo and also to loudness, high pitch and roughness (Pressnitzer et al., 2000; Gomez and Danuser, 2007; Leman, 2007; Van der Zwaag et al., 2011).

#### Music expressions of logic

Moral words produce more legato improvisations than logic words. At this stage we can only speculate about the consistent empirical correspondence between articulation and the representation of logic. A parsimonious interpretation could be as follows: more legato articulation implies more fuzziness and less precision in the perceptual separation of successive notes; we suggest that this may be in correspondence with semantic differences between moral (somehow fuzzier, less discrete, and often more continuous in nature) and logic (discrete, precise, categorically detached from each other) concepts.

We noted before that activity, that measures the dimension of energetic vs. relaxed music, is the only musical dimension typically associated with emotion, that does not differ significantly for positive and negative logic music. This result is consistent with a distinction between basic and refined emotions; the latter, including the noetic emotions (Koriat, 2000) relating to knowledge and truth, and the moral positive emotions such as compassion and sympathy, “are more felt that acted upon, thus do not obviously manifest themselves in overt behaviors like attack, embrace, or flight,” and “may not show pronounced physiological upset” (Frijda and Sundararajan, 2007). Accordingly, the music for this kind of concepts shows lower activity (Figure 1). Instead, moral negative emotions are mainly represented in music perception by anger, a high activity emotion (Zentner et al., 2008).

Several theories of meaning claim that only affections elicited by moral judgments are capable of grounding morality, but that the concept of morality itself cannot be grounded perceptually (Mahon and Caramazza, 2008; Andrews et al., 2009). A different perspective, focusing on the equality component of morality rather than its affective content has been considered, where moral/immoral words are for instance positively associated with visual symmetry/asymmetry (Lakens et al., 2011). While equality is considered a crucial factor in justice and ethics for many philosophers, other inherently asymmetrical principles of social organization are active in moral psychology, such as hierarchy. For theoreticians like Durkheim, Kohlberg, and Shweder, morality tends to preserve social hierarchical order (Rozin et al., 1999). Social hierarchy has been shown to be grounded iconically through force, magnitude, space, and time (Rai and Fiske, 2011). Looking for representation of these symmetry-asymmetry principles in music structure, we analyzed the improvisations' entropy and melodic entropy, which measure symmetry and order in pitch distribution (see Methods). We obtained significant differences in entropy between positive and negative groups (Figure 1, Figure S1 and Table 1): positive concepts are associated to lower entropy music. This indicates that “positive” music is more ordered and more asymmetrical in pitch distribution: there is a stronger tendency for some pitches to occur more frequently than others both in local temporal windows and globally in the improvisation. Therefore “positive” music has a better defined linear hierarchical order based on frequency of note apparition. In music, pitch repetition and an asymmetric hierarchic organization depending on repetition frequency is a fundamental structuring principle in many musical styles, among them Western tonality (Zanette, 2006; Rodriguez Zivic et al., 2013). Melodic entropy has been also shown to have an inverse relation to predictability of melodies (Eerola et al., 2002), that is to an actual realization in the music of cognitive expectations developed by listeners (Meyer, 1961; Krumhansl, 2002). Our results suggest that positive words elicit more predictable music than negative words, although a listening experiment would be needed to corroborate this.

The moral force of music and its influence on human character has been recognized since Antiquity. Asserting that music has both positive and negative effects on morality implies that it is possible to distinguish between the music associated with the concepts of moral and immoral. We found indeed significant differences between the music associated with both categories, and also between the music of truth and of falsehood concepts. Our results support explanations for these differences based on emotions associated to morality and truth, and on metaphorical mappings from the conceptual to the musical domain.

## Methods

### Participants

A total of 19 pianists (5 females, age 37 ± 7 year) participated in the improvisation experiment. They were professional musicians with at least 10 year of musical activity in different backgrounds, from classic to experimental and popular music. A total of 12 participants (8 females, age 27 ± 4 year) with no musical training participated in the control experiment. All the participants signed a written consent form.

The experiments described in this paper were approved by the ethics committee Comité de Ética del Centro de Educación Médica e Investigaciones Clínicas “Norberto Quirno” (CEMIC) qualified by the Department of Health and Human Services (HHS, USA): IRb00001745-IORG 0001315.

### Experiments

The experiment was designed using the Psychtoolbox library running on MATLAB. Participants were instructed to freely improvise on a MIDI keyboard inspired in words appearing on a computer screen.

Once shown the target word, the participant was allowed to play for a maximum of 20 s, before the next word appeared on the computer screen. Improvisations were produced with a Kurzweil K2500XS MIDI keyboard and recorded with the software Sonar 4. We used a piano timbre, library GrandP 2V-32 of the software Reason 3. The loudspeakers were Tannoy Active and we used a Motu Traveler audio interphase. Improvisations were recorded in a soundproof room at LIPM (Laboratorio de Investigación y Producción Musical del Centro Cultural Recoleta, Buenos Aires, Argentina). The complete set of MIDI files is available at Supplementary Materials. The original words in Spanish are: *Positive logic*: verdad, certeza, consistencia, exactitud, adecuación, autenticidad, conformidad, and realidad. *Negative logic*: falsedad, inexactitud, incorrección, inadecuación, error, duda, engaño, and tergiversación. *Positive morality*: lealtad, bondad, caridad, solidaridad, humildad, generosidad, prudencia, and paciencia. *Negative morality*: lujuria, gula, pereza, maldad, avaricia, envidia, vanidad, and ira.

### Quantification of musical parameters

The quantification of the acoustical parameters is standard and can be found elsewhere (Caclin et al., 2005). They were computed using MIRToolbox on the sound files generated from MIDI files using Timidity software. The information-theoretic parameters has been applied to music for quantifying symmetry, order and predictability, and we computed them following (Eerola et al., 2002). Entropy was measured in windows of 7 s, about the estimated size of auditory sensory memory (Fraisse, 1982), with 2 s overlap and then averaged over all windows; the results persist using windows from 5 s to the full length of the improvisation and different overlapping intervals. Finally, the computation of the musical parameters is detailed in Mesz et al. (2011).

### Machine learning model

We used a Gaussian Naive Bayes (NB) classifier trained in a supervised learning setting, normalizing the dimensions with Z-scores (Alpaydin, 2009). We used the *F*_1_ metric as a measure of accuracy of our classifier, defined as the geometric mean between two other measures: *precision* and *recall*. Considering only one label, such as negative moral, *precision* is the ratio between the number of correctly classified instances and all the instances that where classified as negative moral. The *recall* is the ratio between the number of instances that where correctly classified and all instances that are tagged as negative morality in the corpus, regardless how the model classified it. NB was used both for classification based on individual features as well as for the combination of all of the features. To compute the significance of the increase in classification accuracy from the best individual feature to the combination, we estimated the distribution of classification accuracy for different assignments of samples to the training and test sets, resulting in the reported mean accuracy and the standard deviation, from which a *p*-value was computed following a *t*-statistics analysis.

### Music emotion model

This model is implemented in the MIRToolbox for MATLAB which was developed by Lartilot and colleagues. Full details of the model can be found in reference (Lartillot et al., 2007); here we merely summarize the main aspects. A collection of 110 film soundtracks was rated by university students in Likert scales for the three emotion dimensions of valence, activity and tension. The ratings were fitted using Multiple Linear Regression on a set of acoustic and psychoacoustic features, selected for theoretical reasons and extracted from the soundtracks using MIRToolbox; the five best predicting features for each emotional dimension were retained in the model. These features are: for activity, root mean square energy of the signal, fluctuation peaks, spectrum centroid, spectrum spread and spectrum entropy; for valence, root mean square energy, fluctuation peaks, key clarity, mode and spectrum novelty; for tension, root mean square energy, fluctuation peaks, key clarity, harmonic change and novelty of the chromagram (distribution of energy along pitches).

### Quantification of musical parameters

The quantification of the acoustical parameters is standard and can be found elsewhere (Caclin et al., 2005). They were computed using MIRToolbox on the sound files generated from MIDI files using Timidity software. The information-theoretic parameters have been applied to music for quantifying symmetry, order and predictability, and we computed them following (Eerola et al., 2002). Note that we computed pitch-class entropy: we consider not actual notes but pitch classes (that is, all the C's in the keyboard are counted as the same MIDI note 60, in spite of the fact that their MIDI note numbers differ in multiples of 12. There are 12 pitch classes corresponding to the notes of the chromatic scale). Entropy was measured in windows of 7 s, about the estimated size of auditory sensory memory (Fraisse, 1982), with 2 s overlap and then averaged over all windows; the results persist using windows from 5 s to the full length of the improvisation and different overlapping intervals. Finally, the computation of the musical parameters is detailed in Mesz et al. (2011).

### Conflict of interest statement

The authors declare that the research was conducted in the absence of any commercial or financial relationships that could be construed as a potential conflict of interest.
