# Supplementary material for: The music of morality and logic
Source: Front Psychol. 2015 Jul 1;6:908. doi: 10.3389/fpsyg.2015.00908 (PMC4486752; doi:10.3389/fpsyg.2015.00908)
Supplement: Supplementary file 1 [file DataSheet1.PDF]

LSA analysis for moral and logic domains

The concepts used in our study may have an overlapping mental representation, such as may be the case, for instance, with ‘good’ and ‘truth’. A significant overlap may be a confounding factor in our analysis, as the ability of music to convey concepts associated with ‘good’ could explain why it can also convey information related to ‘truth’. While a complete elucidation of this concern lies beyond the scope of the present manuscript, we partially addressed it using a well-established statistical technique, Latent Semantic Analysis (LSA). LSA creates a vectorial representation of concepts based on their frequency of co-occurrence in large text corpora, in such a way that the semantic relatedness of concepts can be estimated by the proximity of their respective vectors [1]. We used 300-dimensional vectors obtained by training with the TASA database [2] to represent the 8 concepts in each of the positive morality, negative morality, positive validity and negative validity classes. We then applied the following rationale: if the semantic content of any of these two classes overlaps, the corresponding space spanned by their semantic vectors should also be significantly large. In order to measure this, we proceeded as follows: for each class, we computed a measure of the volume spanned as

$$V_i = \left| det\, X_i X_i^T \right|^{1/8}$$

where $V_i = \{\boldsymbol{c}_{1,i}, \boldsymbol{c}_{2,i}, \ldots, \boldsymbol{c}_{8,i}\}$ is the set of 8 semantic vectors in the class. We computed the inter-class volume for each pair as

$$V_{ij} = \left| det\, X_i X_j^T \right|^{1/8}$$

Finally, to obtain a null hypothesis, we estimated the expected values for V_i and V_{ij} from 100 random samples of LSA vectors, and from random 300-dimensional vectors (in all cases the vectors have norm 1). The results in Fig. S1 show that the space spanned by the overlap across concept classes (red) is significantly smaller than the space corresponding to the intra-class concepts (blue). Some of the cross-class spaces (such as positive and negative morality, ‘g’ in the figure) are larger than what would be expected from the null hypothesis (‘m’ and ‘n’), but still quite smaller than the intra-class spaces (‘a’ and ‘b’). These results indicate that, to the extent that semantic relatedness is captured by LSA, the overlap across concept classes is minimal.

[1] Deerwester, S., Dumais, S. T., Furnas, G. W., Landauer, T. K., and Harshman, R. (1990). Indexing by latent semantic analysis. *J. Am. Soc. Inf. Sci.* 41, 391–407.

[2] Diuk, Carlos G., D. Fernandez Slezak, I. Raskovsky, M. Sigman, and G.A. Cecchi. A quantitative philology of introspection. *Frontiers in integrative neuroscience* 6 (2012).

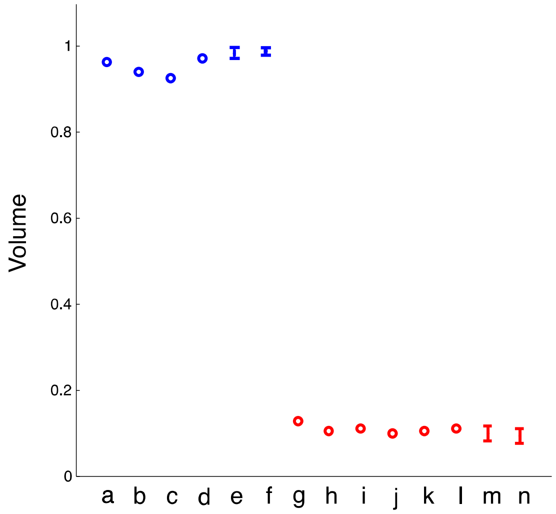

Figure S1. Estimation of the semantic volume spanned by concepts associated with positive morality (a), negative morality (b), positive validity (c), negative validity (d), matching random samples from LSA-TASA (d), random vectors (f), the overlap between positive and negative morality (g), positive morality and negative validity (h), positive morality and positive validity (i), negative morality and positive validity (j), negative morality and negative validity (k), positive validity and negative validity (l), samples from random LSA vectors (m) and from random vectors (n).

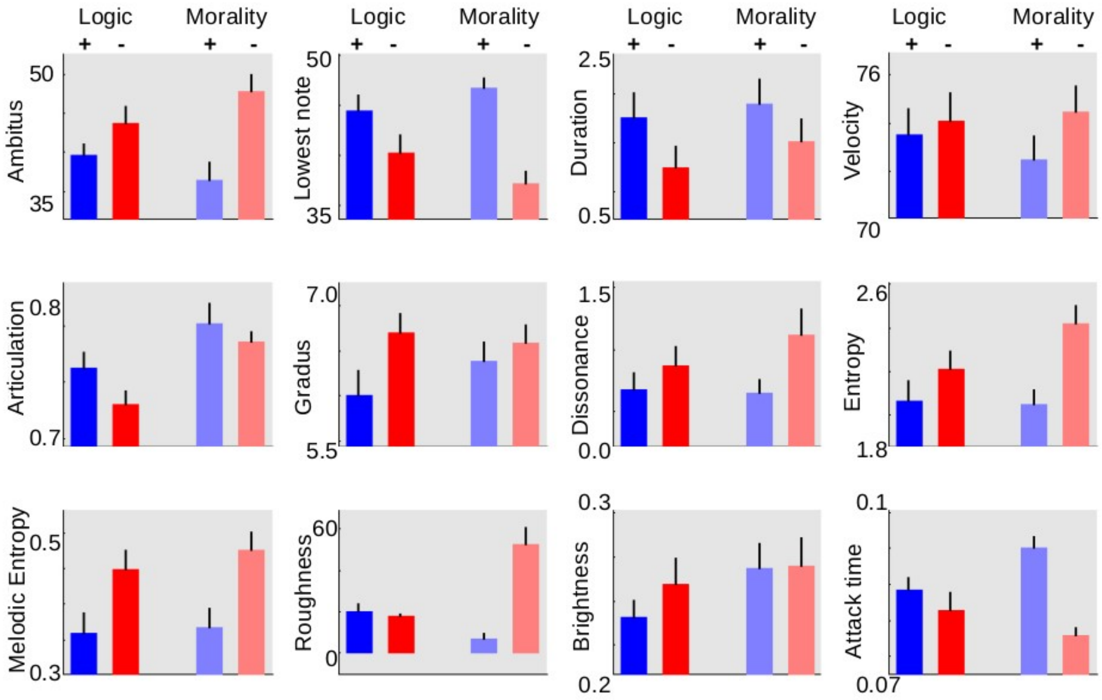

Figure S2. Mean values and standard deviations averaged across words and pianists for the parameters not shown in Figure 2.
